# Q methodology as an integrative approach: bridging quantitative and qualitative insights in a mixed methods study on mathematics teachers’ beliefs

**DOI:** 10.3389/fpsyg.2024.1418040

**Published:** 2024-07-12

**Authors:** Nils Buchholtz, Maike Vollstedt

**Affiliations:** ^1^Faculty of Education, University of Hamburg, Hamburg, Germany; ^2^University of Bremen, Faculty 3 – Mathematics and Computer Science, Bremen, Germany

**Keywords:** Q methodology, Q sort, Likert, beliefs, pre-service teachers, mathematics, mixed methods

## Abstract

**Introduction:**

This study explores the beliefs of pre-service mathematics teachers regarding the teaching and learning of mathematics.

**Methods:**

We employed a mixed methods approach, combining quantitative Likert-scale surveys and Q methodology, an integrative quantitative and qualitative approach. A sample of 33 pre-service teachers participated in the study. Initially, Likert-scale surveys were used to ascertain general trends in belief orientations. Subsequently, the same participants engaged in a Q sort exercise, which allowed for a nuanced exploration of individual belief systems by prioritizing the same survey statements within a forced-choice grid. Qualitative interviews further enriched these findings.

**Results:**

Q methodology analysis identified distinct belief profiles, characterized by their prioritization of specific educational practices and the contextual and subjective interpretations that underpin these preferences. The qualitative interviews provided deeper insight into the reasoning behind participants’ choices in the Q sort, illustrating the complex, sometimes contradictory nature of personally held beliefs that traditional Likert-scale approaches may obscure.

**Discussion:**

Our results underscore the utility of combining Q methodology with conventional teacher belief survey techniques to achieve a more holistic understanding of pre-service teachers’ beliefs. This approach reveals the complexity within individual belief systems and highlights the potential for mixed methods research to refine the measurement and interpretation of psychological constructs in educational settings.

## 1 Introduction

Teacher beliefs about the teaching and learning of mathematics play a central role for mathematics teachers’ instructional actions in the classroom. They represent an affective component of mathematics teacher competence and are, thus, assumed to have an influence on their interaction with the world, i.e., also in their classroom (Philipp, 2007). Typically, in empirical psychological studies in mathematics education, beliefs are understood as personality traits and are methodically investigated quantitatively via Likert scale-based surveys. In these, items are formulated in statements and the degree of individual agreement to items is measured (e.g., Laschke and Blömeke, 2013; Voss et al., 2013). Correspondingly, researchers utilize primarily quantitative data evaluation techniques such as exploratory and confirmatory factor analyses to unveil belief-dimensions as hidden patterns in the items influencing respondents’ beliefs. One notable application of this approach was for example in the international TEDS-M study and its follow-up studies that analyzed (future) mathematics teachers’ professional competence, and also surveyed teacher beliefs on the nature of mathematics and the teaching and learning of mathematics (Tatto et al., 2008; Wang and Hsieh, 2014; Yang et al., 2020).

However, there is criticism with respect to the application of Likert-scale surveys in the study of teachers’ beliefs. Among other criticisms, it is commonly noted that items can be answered independently of each other (Carifo and Perla, 2007) and that the situation-specific context of teaching is usually not included in the formulation of items (Seifried, 2009). This may then obscure teachers’ differentiated interpretation of the items (Safrudiannur and Rott, 2019, 2020, 2021). Thus, when answering, it is hardly possible for the teachers to express their subjective and individual interpretation of the items, which is related to their actual classroom experience. This then may affect the validity of the results that are obtained by the use of respective instruments (Aeschbacher and Wagner, 2016; Biedermann et al., 2016). However, these context-specific interpretations of items are especially relevant for the study of mathematics teachers’ beliefs when it comes to the question how they are related to teachers’ expertise and their actual teaching practice, as this relationship is of high complexity and still not clear. For example, when examining pre-service teachers who are still in teacher training, it can be assumed that they perceive or interpret the wording of a Likert item such as “Students learn mathematics best by attending to the teacher’s explanations” differently than participants with a long professional experience and a lot of teaching practice. While the latter may be able to weigh up the meaning of different items situationally and are able to also cognitively process contradictory beliefs (Furinghetti and Pehkonen, 2002), the former may tend to make absolute assessments because each item has to be answered largely independently of their experience (Seifried, 2009; Buehl and Beck, 2015). Hence, in response to the criticism concerning measuring teacher beliefs, innovative research methods (and instruments) are needed in mathematics education research in which Likert-items can be discussed and related to each other so that the examination of teachers’ subjective beliefs in more detail is possible and the complexity of the research object can be adequately addressed.

In this study, we therefore use a methodological approach that addresses this issue. Q methodology offers a nuanced mixed methods research technique that integrates both quantitative and qualitative approaches that can be used to measure teachers’ beliefs more subjectively and allows researchers to identify and explore different patterns of thought within a group (Stephenson, 1953; Brown, 1980; Stenner and Stainton-Rogers, 2004; Newman and Ramlo, 2010; Ramlo, 2016). In our study, which is part of a Special Issue in Frontiers on Best Practice Approaches for Mixed Methods Research in Psychological Science, we show how we used Q-methodology to elicit the subjective beliefs of 33 prospective mathematics teachers (M. Ed.) on existing Likert-scale items that measure beliefs about the teaching and learning of mathematics. We chose an integrated mixed methods approach and analyzed the participants’ patterns of thought both quantitatively and qualitatively. The aim of the study is to be able to make more precise statements about the belief patterns of pre-service teachers than would have been possible using only a quantitative Likert-scale evaluation. To this end, we specifically highlight the strengths of our approach and the extent to which the mixed method approach provides added value to the survey of mathematics pre-service teachers’ beliefs.

## 2 Theoretical background

### 2.1 Teacher beliefs

Teacher beliefs are one of the central affective components of teachers’ professional competence (Shulman, 1986; Bromme, 1997; Seifried, 2009). As Dohrmann (2021) states, Pajares (1992) has aptly stated that whenever we speak of “teachers’ beliefs” in general, we are always referring to teachers’ beliefs about school, teaching, learning and students. Pajares (1992) wrote his well-known essay, in which he spoke about teachers’ beliefs as a “messy construct” over 30 years ago. However, up to date, the various sub-disciplines of psychological and educational science, that deal with teachers each use their own terms for teachers’ beliefs. Dohrmann (2021) describes that the terms used to describe teachers’ beliefs range from “views” (Seifried, 2009) to “conceptions” in subject-didactical research (Hartinger et al., 2006). In studies on teachers, terms such as “subjective theories” (Martínez et al., 2017) or “educational beliefs” (Pajares, 1992; König, 2012) are used. “Teachers’ beliefs,” on the other hand, appear in research on teachers (Turner et al., 2009; Fives et al., 2015) as well as in subject didactical research (Thompson, 1992; Philipp, 2007).

This plethora of terms makes it obvious that despite extensive research on teachers’ beliefs, especially in the context of pedagogical-psychological oriented approaches, a precise and universally accepted definition of beliefs remains elusive, underscoring the complexity of this concept within the educational field (Törner, 2002; Leder, 2019). Philipp (2007) defines beliefs as “the lenses through which one looks when interpreting the world” (p. 258). Richardson (1996) proposes a domain-unspecific definition of beliefs that is based on a broader understanding. She understands beliefs to be “psychologically held understandings, premises, or propositions about the world that are felt to be true” (Richardson, 1996, p. 103). This refers to a person’s epistemological stands towards an object—it’s worldviews—, which includes affective attitudes and the readiness to act (Grigutsch and Törner, 1998) and which, in contrast to knowledge, are dependent on the degree of individual agreement (Beswick, 2005, 2007). These definitions suggest that beliefs encompass both cognitive and affective dimensions, influencing how individuals perceive and interact with their environment (Furinghetti and Pehkonen, 2002). Unlike professional knowledge, which is consensual, beliefs can vary significantly among individuals (Beswick, 2005; Philipp, 2007).

### 2.2 The fluidity and yet possible fixed nature of teacher beliefs and how they influence teaching practice

Teacher beliefs contain both conscious and unconscious elements, hypotheses and expectations. These beliefs are interconnected and influence each other. Some beliefs are more central and more firmly anchored than others (Furinghetti and Pehkonen, 2002). Centralized beliefs are considered to be relatively fixed in nature and to be only changeable in the longer term within the scope of training processes, cumulative experiences or through radical changes based on key experiences (Beijaard and De Vries, 1997; Reusser et al., 2011), although it is not yet sufficiently clear which factors influence their changes in the development of expertise (Biedermann et al., 2015) and research yielded contradictory results so far (Dohrmann, 2021). So, with regard to the long-term development of beliefs, it is assumed that they are relatively stable with respect to restructuring, and to a certain extent can act as psychological filters and/or barriers (Reusser et al., 2011; Fives and Buehl, 2012). On the other hand, more peripheral beliefs are easier to adapt to new information and are closely linked to the development of expertise (Beijaard and De Vries, 1997; De Vries et al., 2014). So, beliefs can change in teachers’ professional development (Swars et al., 2009; Eichler and Erens, 2015) or can be influenced by the abilities of students that are taught (Zohar et al., 2001; Beswick, 2018; Safrudiannur and Rott, 2019).

It is also assumed that beliefs are organized into an individual’s belief systems, which are grouped around an object in the sense of an overarching affect, and in which different and even contradicting beliefs can coexist (Furinghetti and Pehkonen, 2002; Leder et al., 2002; Philipp, 2007). For example, a teacher may simultaneously believe that understanding mathematical concepts is more important than memorizing them, but still use many routine exercises in the classroom (Furinghetti and Pehkonen, 2002). This relativistic form, and especially the possibility of the coexistence of conflicting beliefs, make beliefs as an intrapsychic construct, with varying degrees of affective and cognitive components, particularly important when they become relevant in the complex context of teaching. This variability suggests that teachers may hold different beliefs depending on the situational context (Safrudiannur and Rott, 2019), challenging the notion of a direct and straightforward link between beliefs and behavior, such as in the enactment of teaching practices (Dohrmann, 2021).

Research on teacher action assumes a significant link between a teacher’s actions in situational contexts and corresponding subjective beliefs (Thompson, 1992; Calderhead, 1996; Schoenfeld, 1998; Felbrich et al., 2010; Schmotz et al., 2010). It is proposed that beliefs orient and guide action, highlighting their fundamental role in teaching practices. This assumption is known as the consistency assumption (Grigutsch and Törner, 1998). Yet, the current state of research with respect to the relationship between teachers’ beliefs and their teaching practices is not clear either: Some researchers have shown consistent beliefs and practices (e.g., Staub and Stern, 2002; Safrudiannur and Rott, 2017) whereas others have shown inconsistencies (e.g., Furinghetti and Pehkonen, 2002; Li and Yu, 2010; Cross Francis, 2015). One possible interpretation of these inconsistencies is that teachers may hold varying beliefs depending on the situation or context in question (Safrudiannur and Rott, 2020, 2021) or a different emotional involvement of teachers in action situations (Seifried, 2009). A lack of experience or external factors, such as support from school management or educational policy guidelines, can also lead to inconsistencies between teachers’ beliefs and actions (Buehl and Beck, 2015).

### 2.3 Teachers’ beliefs with regard to the teaching and learning of mathematics

Despite the complex nature of teacher beliefs and how they may or may not affect teachers’ actions, there is agreement that beliefs can be domain-specific (Törner, 2002; Eichler and Erens, 2015) or even situation-specific (Schoenfeld, 2010; Kuntze, 2011). For mathematics teachers, there is a broad consensus in the literature on the differentiation of profession-related beliefs. Ernest (1989, p. 250) proposes three views of mathematics: the relatively static instrumentalist view (mathematics is seen as an accumulation of facts, rules, and skills to be used in pursuit of some external end), the also rather static Platonist view (mathematics is seen as a static but unified body of certain knowledge), and the more dynamic problem-solving view (mathematics is seen as a process of inquiry and knowledge, not a finished product, for its results remain open to revision). Ernest (1989) also identifies these views with beliefs about teaching and learning mathematics. The instrumentalist view sees the teacher as an instructor who helps students master mathematical skills correctly, the Platonist view sees the teacher as an explainer who helps students understand mathematical concepts, and the problem-solving view sees the teacher as a facilitator who helps students construct knowledge. Other studies also identify these beliefs about the acquisition of mathematical knowledge or the teaching and learning of mathematics as a significant dimension of mathematics teachers’ epistemological beliefs (Furinghetti and Pehkonen, 2002; Staub and Stern, 2002; Handal, 2003; Kuntze, 2011). In different studies from research on teachers’ professional competence, transmission-oriented beliefs, in which students are viewed as passive recipients of knowledge (e.g., “Students learn mathematics best by attending to the teacher’s explanations.”), are distinguished from constructivist-influenced beliefs that endorse the principles of constructive learning (e.g., “Teachers should encourage students to find their own solutions to mathematical problems even if they are inefficient.”) (Peterson et al., 1989; Staub and Stern, 2002; Laschke and Blömeke, 2013; Voss et al., 2013). Although the question of how teacher beliefs influence student achievement is far from conclusive, it is proposed that dynamic beliefs about mathematics and constructivist teaching-learning approaches are more strongly related to an emphasis on procedural, iterative mathematics in instructional settings (Reusser et al., 2011).

### 2.4 Studies on the relationship between mathematics teachers’ beliefs and expertise

Studies on pre-service teachers in mathematics educational research consistently show that pre-service teachers usually already have initial beliefs about teaching and learning of mathematics at the beginning of their studies, which also influences the acquisition of their professional knowledge. In several studies, mathematics pre-service teachers therefore exhibit a strong tendency towards constructivist teaching-learning beliefs and a strong rejection of transmission-oriented beliefs (Schmeisser et al., 2013; Buchholtz and Kaiser, 2017). These beliefs are usually shaped by their own school experience as a learner of mathematics and the experiences of the university entry phase (Blömeke et al., 2014; Buchholtz and Kaiser, 2017). Constructivist beliefs about the teaching and learning of mathematics then increase throughout the entire teacher training while transmission-oriented beliefs stagnate even more. Prospective teachers within the TEDS-M study also showed strong constructivist beliefs (Felbrich et al., 2010). Pre-service teachers usually lack own teaching experiences at the beginning of their studies, but gain first teaching experiences during their years of studies, for example in internships, which can challenge their beliefs and lead to changes in their belief systems (König et al., 2022).

Studies described by Safrudiannur and Rott (2019) emphasize this impact of teaching experiences on the beliefs and practices of teachers. Page and Clark (2010) illustrate that a teacher’s experiences, both in teaching mathematics and as a mathematics learner, significantly influence their beliefs and instructional practices. Similarly, research by Huang et al. (2010) shows a distinct contrast between novice and experienced teachers in China, where novices prioritize effective teacher guidance, while experienced teachers focus on fostering students’ mathematical and higher-order thinking skills. Conversely, larger-scale studies present conflicting results. For instance, Safrudiannur and Rott (2020) highlight that teaching experience, typically quantified by years, may not impact teachers’ beliefs and practices as previously thought. Nisbet and Warren (2000) found no significant link between the duration of teaching and teachers’ beliefs about mathematics instruction. Drageset (2010) observed that the number of teaching years does not correlate with beliefs regarding the importance of rules, correct answers, reasoning, or justification in teaching mathematics. Additionally, Wilkins (2008) reported that while teaching years have no direct effect on didactic beliefs and practices, there is an indirect negative effect. Similarly, Ren and Smith (2018) identified no association between years of teaching and either student-centered or teacher-centered beliefs. Beswick (2005) also noted a negative correlation between years of teaching experience and teachers’ beliefs in a smaller study.

If we look on how teacher beliefs influence teaching practice, the focus of studies is more on in-service teachers. Empirical evidence from the German COACTIV study with in-service teachers has revealed a significant relation between constructivist-influenced beliefs about teaching and learning mathematics and the quality of teaching as well as student learning outcomes (Voss et al., 2013). However, these findings complement older findings that found stronger transmission-oriented beliefs among practicing teachers, whereby teachers with transmissive learning beliefs designed lessons in a less challenging and activating way, rather avoiding mistakes (Dubberke et al., 2008). Overall, the findings on beliefs among practicing teachers therefore do not seem clear (Reusser et al., 2011). Although Voss et al. (2013) found a strong negative correlation between them, there does not seem to be a fundamental contradiction between constructivist-influenced and transmission-oriented beliefs. Instead, the results of the COACTIV-study point to a crucial functional balance between different beliefs of teachers in varying teaching-learning contexts (Voss et al., 2013). It may therefore be advantageous under certain conditions or for specific groups of students if, on the basis of a constructivist orientation, transmissive beliefs are nevertheless accepted to a certain extent (Voss et al., 2013). Depending on the teaching situation, different beliefs may be relevant to teachers’ professional actions, and beliefs therefore depend heavily on subjective judgements of the situation. Jaschke’s (2018) findings in a study, that based on Q methodology, support the assumption of subjective situation-specific beliefs. In his study with mathematics pre-service teachers, he finds two types of teachers, one of which shows a rather dichotomous orientation towards a constructivist-influenced belief and rejects transmissive-oriented beliefs, while the second shows agreement on both beliefs and shows a more mixed profile that integrates both constructivist-influenced and transmissive-oriented beliefs.

### 2.5 Different methods to investigate beliefs

If one looks at previous approaches to the empirical survey of beliefs in the field of teacher competence research from a methodological perspective (even beyond these studies), beliefs are primarily collected quantitatively using item rating procedures. One widely used method is the statistical evaluation of Likert scale surveys using exploratory and/or confirmatory factor analyses. These methods are generally used in studies with larger sample sizes to investigate the professional competencies of teachers, such as TEDS-M (Laschke and Blömeke, 2013) and COACTIV (Kunter et al., 2011), but also by Carter and Norwood (1997). This approach facilitates the comparison of individuals in relation to well-defined constructs and proves to be effective for the study of long-lasting beliefs or perspectives. In general, this approach to surveying teachers’ beliefs offers the possibility of collecting large amounts of data with little effort and subsequently evaluating it quantitatively.

However, the survey method can conceal context- and subject-related forms of expression of beliefs, when surveying teachers in different expertise groups. For example, in traditional Likert scale surveys, teachers with different levels of expertise may rate the items differently (Safrudiannur and Rott, 2019). They found that teachers that teach in classes with many low-achieving students prioritize items differently than those teaching in classes with many high-achieving students, the former prioritizing more instrumental beliefs and the latter prioritizing more dynamic beliefs. Furthermore, there is evidence that context-independent Likert-based assessment procedures favor socially desirable responses in terms of prioritizing constructivist beliefs (Di Martino and Sabena, 2010; Aeschbacher and Wagner, 2016; Safrudiannur and Rott, 2020, 2021). In addition, Likert scale surveys often present items independently of context, so that the individual statements do not have to be related to each other (Carifo and Perla, 2007; Seifried, 2009). From the point of view of researching beliefs in different expertise groups (e.g., pre-service teachers vs. in-service teachers) or with reference to different contexts, the question therefore arises of context- and subject-related survey methods of beliefs that go beyond previous approaches aimed at persistent constructs.

On the other hand, purely qualitative methodological approaches also exist in more educational research on the beliefs of (mathematics) teachers. They can represent an alternative to the application of Likert-scale surveys as they can take much greater account of the subjectivity of the interviewees. Generally, teachers are interviewed about their beliefs (e.g., Gräsel et al., 2006; Harteis et al., 2006; Hirsch and Buchholtz, 2023), which are then usually reconstructed qualitatively. However, the methodological disadvantage here is that the questions with which the interviewees are asked to articulate their beliefs are usually quite open, which can lead to difficulties in the comparability of reconstructed beliefs or difficulties to relate the obtained results to previous research findings. In addition, the reconstructions can also be highly dependent on the interpretation of the researcher. This also poses challenges for the trustworthiness of the results obtained with regard to the methodological instrument of data collection.

From a methodological perspective, both strengths and weaknesses can be identified in the investigation of mathematics teachers’ beliefs in both quantitative and qualitative approaches. Due to their subjectivity, beliefs are also highly complex and extremely difficult to measure methodologically. It therefore seems promising to pursue integrative approaches within the framework of mixed methods research designs and to develop innovative instruments that integrate quantitative and qualitative measures (Johnson and Onwuegbuzie, 2004; Gläser-Zikuda et al., 2012).

### 2.6 Q methodology as a mixed methodology and its utility in measuring teacher beliefs

One such mixed method measurement approach that has already been used to investigate mathematics teacher beliefs (Jaschke, 2017, 2018; Lim-Ratnam et al., 2022) is Q methodology (Müller and Kals, 2004; Newman and Ramlo, 2010; Coogan and Herrington, 2011). Q methodology, already introduced by Stephenson (1953) and further elaborated by Brown (1980), is a less frequently used methodology in mathematics educational and psychological research, but is particularly useful when studying beliefs as it studies human subjectivity. Researchers can explore different ‘points of view’ on a particular issue among groups of people (Coogan and Herrington, 2011), as Q studies investigate correlations between individuals, rather than items. This methodology does not impose predetermined meanings on participants. Instead, participants determine what holds meaning and significance from their own perspectives through a process called Q sorting, where they rank belief statements on a range. The data collected from multiple individuals is then subjected to a quantitative inverse factorial analysis in order to identify shared belief systems or perspectives (i.e., different types of sorts based on persons, not items), revealing groups of people who have ranked statements in a similar order. This yields a set of factors represented by all the presented statements configured in distinct and characteristic ways, rather than different subsets of the statements. The interpretation and significance of these configurations are attributed *a posteriori* through analysis, rather than predetermined assumptions. Different participants may interpret a statement differently, for instance, one person may perceive a transmission-oriented statement as negative, while another may interpret it as a positive statement, especially in contexts like teaching students with learning difficulties (Coogan and Herrington, 2011).

The main aim of Q methodology is therefore to form types of subjective views of a certain subject area that reveal similarities and differences in subjective construction (*cf.* Müller and Kals, 2004). While traditional Likert-scale questionnaires require large samples for statistical significance, Q methodology can yield meaningful insights with a small, targeted sample (Ernest, 2011; Ho, 2017). Typically, Q methodology works with 10 to 50 participants (Brown, 1980). In addition to Q sort data, individual comments and explanations provided during the Q sort process give context and depth to these shared perspectives when analyzed qualitatively, offering a richer and even more nuanced understanding of participants’ beliefs (Brown, 1980 McKeown and Thomas, 1988). While traditional surveys often aggregate data to find common trends, Q methodology focuses on the subjective experiences and interpretations of individuals, making it a valuable tool in educational and psychological research. Q methodology has been successfully applied in empirical studies on teachers’ beliefs (Rimm-Kaufman et al., 2006; Ernest, 2011; Jaschke, 2017, 2018; Lundberg, 2019; Lim-Ratnam et al., 2022).

This integration of quantitative analysis and qualitative insights (Johnson and Gray, 2010) offers a comprehensive view of the complex interplay of beliefs within a certain group. This combination makes Q methodology as a mixed methodology particularly powerful, as it bridges the gap between numerical data and human narrative, ensuring that the subjectivity of personal considerations of participants and the context sensitivity of beliefs are preserved in the quantitative analysis (Stenner and Stainton-Rogers, 2004; Ridenour and Newman, 2008; Ramlo and Newman, 2011). This integrative mixed methods approach to studying teacher beliefs combines the specific strengths of individual methodologies and compensates for their methodological weaknesses (Johnson and Onwuegbuzie, 2004; Johnson and Christensen, 2017; Schoonenboom and Johnson, 2017).

## 3 Research design and methodology

### 3.1 Research questions

Accordingly, in this study, we follow the approach of Jaschke (2017, 2018) and consider how mathematics pre-service teachers’ beliefs about mathematics teaching and learning can be analyzed using Q methodology. In an integrated approach, we combined a quantitative standardized Likert scale questionnaire survey on beliefs with a Q sort of the same items and interviewed the participants after their Q sort about the perceived similarities and differences between the two quantitative approaches and their subjective perception of the statements, thus also enabling qualitative aspects of Q methodology. Both quantitative and qualitative data collection were integrated into a quantitatively driven concurrent-sequential (quan + QUAN) → qual mixed methods research design (Morse, 1991; Schoonenboom and Johnson, 2017). The rationale for this design is twofold: first, to clarify the importance of Q methodology in the study of beliefs and to explore its advantages over Likert scale methods (which is why these quantitative findings are prioritized – which in mixed methods designs is indicated with the “quan” in capitals); and second, to highlight the subjective nature of pre-service teachers’ beliefs by complementing quantitative findings with qualitative findings (Greene et al., 1989; Kelle and Buchholtz, 2015). We sought to answer the following research questions:

RQ1 (quan): What types of beliefs about the teaching and learning of mathematics are found among pre-service teachers using the Likert scales, and how can they be characterized?

RQ2 (QUAN): What types of beliefs about the teaching and learning of mathematics are found among pre-service teachers using the Q-method, and how can they be characterized?

RQ3 (QUAN → qual): What insights on the types of beliefs from the Q-sort can be drawn from the different prioritization of items in the Q-sorts with respect to the subjective evaluation of the items?

RQ4 ((quan + QUAN) → qual): What insights on the preferences of the methods to survey beliefs (Likert scales vs. Q-method) that can be drawn from the interviews?

### 3.2 Sample and methods of data collection

In our study, we examined a convenient sample of *N* = 33 mathematics pre-service teachers at the end of their studies (M. Ed. Mathematics for Primary or Secondary School) at the Universities of Bremen (29 pre-service teachers) and Hamburg (4 pre-service teachers), two urban universities in Northern Germany. In this survey, the group of pre-service teachers shows a diverse study background: the majority (26 pre-service teachers) were studying teaching mathematics for upper secondary level, 6 pre-service teachers were studying mathematics for primary level. One participant studied for a teaching degree for special education with subject mathematics and a profile for upper secondary level. All of the pre-service teachers had several months of practical teaching experience at school in internships, so that they were able to relate the statements in the items to concrete teaching experiences in the classroom.

Each participant of the study was surveyed individually, resulting in 33 sessions that were planned as lasting about 45 min each. The data were collected in a three-step process: first, the participants completed the Likert scale questionnaires (5–10 min), second, right after this, the Q sorts were created using the Q sort technique (free in time), and third, an immediate interview of about 10–20 min was conducted on the Q sorts and questionnaires.

At step one, the pre-service teachers were completing the questionnaire containing 14 statements on beliefs about teaching and learning mathematics from the TEDS-M study, where all statements are visible at the same time but are answered independently (Peterson et al., 1989; Laschke and Blömeke, 2013). The instrument has already been tested in previous national and international empirical studies with larger samples of pre-service and in-service teachers (Tatto et al., 2008; Wang and Hsieh, 2014; Blömeke et al., 2015), and several studies identified two scales within the set of statements by exploratory and confirmatory factor analyses (Buchholtz et al., 2013; Buchholtz and Kaiser, 2017; Yang et al., 2020). On the one hand, a transmission-oriented scale on learning mathematics through teacher instruction (eight statements) and, on the other hand, a constructivist-influenced scale on learning mathematics through active learning (six statements). The statements were to be answered on a 6-point Likert scale (1 = “strongly disagree” to 6 = “strongly agree”). Table 1 shows the statements used along with their enumeration based on the TEDS-M instrument (Laschke and Blömeke, 2013). The enumeration was used to refer to specific items later in the data collection.

**Table 1:** Transmission-oriented and constructivist-influenced scales to assess beliefs on the teaching and learning of mathematics (Laschke and Blömeke, 2013).

Transmission-oriented scale	Constructivist-influenced scale
1. The best way to do well in mathematics is to memorize all the formulas.	7. In addition to getting a right answer in mathematics, it is important to understand why the answer is correct.
2. Pupils need to be taught exact procedures for solving mathematical problems.	8. Teachers should allow pupils to figure out their own ways to solve mathematical problems.
3. It does not really matter if you understand a mathematical problem, if you can get the right answer.	11. Time used to investigate why a solution to a mathematical problem works is time well spent.
4. To be good in mathematics you must be able to solve problems quickly.	12. Pupils can figure out a way to solve mathematical problems without a teacher’s help.
5. Pupils learn mathematics best by attending to the teacher’s explanations.	13. Teachers should encourage pupils to find their own solutions to mathematical problems even if they are inefficient.
6. When pupils are working on mathematical problems, more emphasis should be put on getting the correct answer than on the process followed.	14. It is helpful for pupils to discuss different ways to solve particular problems.
9. Non-standard procedures should be discouraged because they can interfere with learning the correct procedure.	
10. Hands-on mathematics experiences aren’t worth the time and expense.	

**Figure 1:**
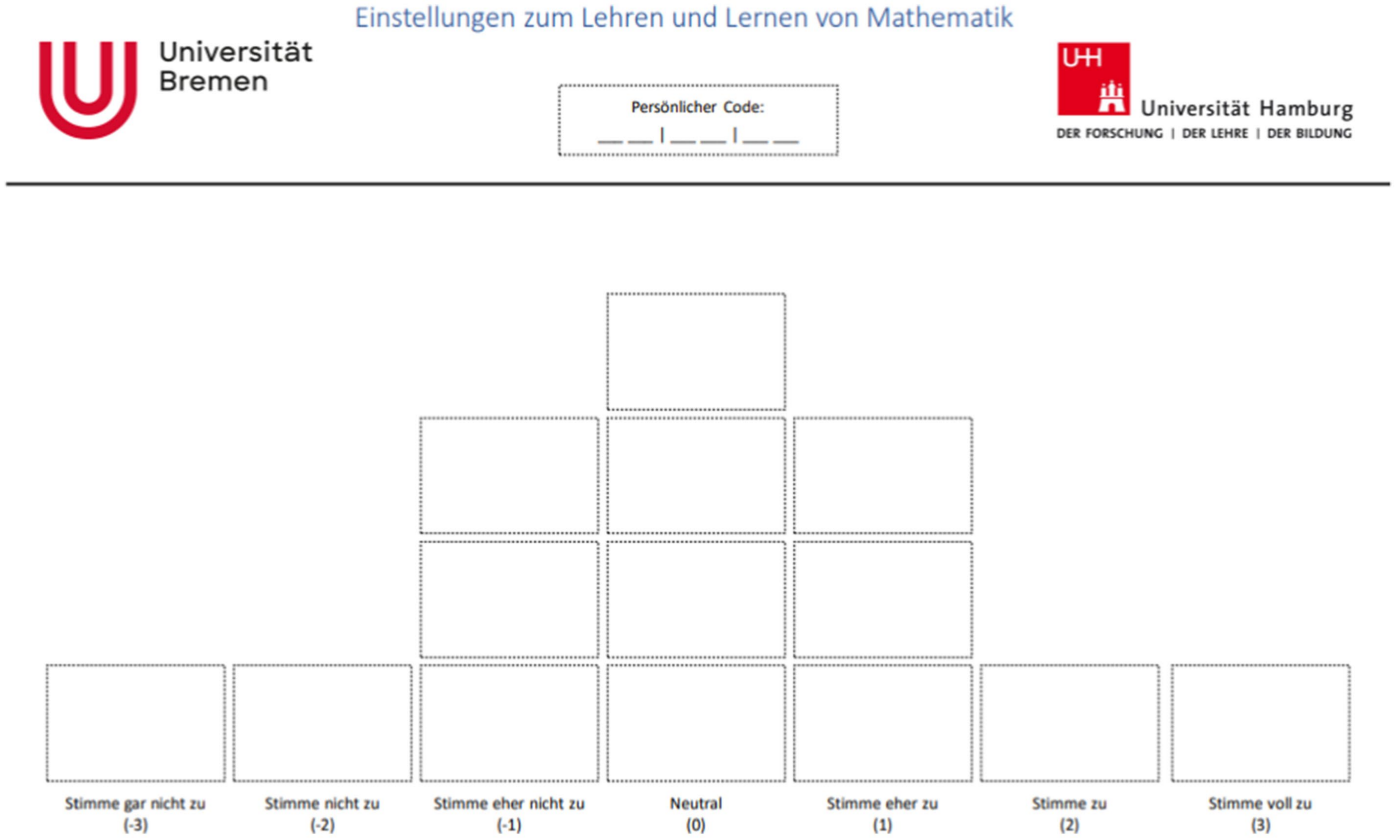
Q grid for the Q sort on beliefs on the teaching and learning of mathematics.

The second step of data collection took place immediately after the pre-service teachers had completed the questionnaires. They were presented with an unsorted Q set containing the same statements and the corresponding Q grid on a poster (see Figure 1). Traditionally in a study applying Q methodology, participants sort a set of statements that are specifically selected and culled for the Q study (McKeown and Thomas, 2013). In this study, to allow a comparison between the research approaches the 14 statements from the TEDS-M were used as Q set. The cards were numbered in the same way as in the questionnaire. The pre-service teachers were instructed to place the statements on the forced Q grid along the scale from (−3) to (3) according to their personal prioritization. There was only room for one statement at position −3 being assigned the lowest level of agreement (“strongly disagree”) and for one statement at position 3 being assigned the highest level of agreement (“strongly agree”) (see Figure 1). The distribution of cards roughly follows a normal distribution, but generally can also take any other non-bizarre symmetrical form (Brown, 1980; Watts and Stenner, 2012). As this was a forced Q sort, only one card could be placed in each cell, and the cards could be swapped and repositioned in between. Due to the limited number of free spaces for each level of agreement–a distinction between forced and so-called unforced Q sorts, where any number of cards can be placed at any level of agreement–the participants had to engage intensively with their own beliefs in order to judge their (dis)agreement with the statements in relation to the other statements (Fisseler, 2023).

Collecting additional qualitative data can help in the interpretation of the final results of Q sorts (Müller and Kals, 2004; Watts and Stenner, 2012; Ho, 2017). Thus, in step three of the data collection, an interview was conducted with each participant following the Q sort. Questions were asked about the individual placements, the context, and the two approaches (Likert questionnaire vs. Q sort). Examples of questions from the interview guide that address individual placements are “Why does card X on the Q grid differ from the questionnaire, here you chose x as the level of agreement?” or “You agreed more/less with card X on the questionnaire than with card Y, with the Q grid it is the other way around–can you explain why?.” Questions were also used to determine the context in which participants agreed or disagreed with the statements. The focus here was on whether individual students or whole learning groups were used as a reference when responding. At the end of the interview, questions were asked on a meta-level about the two approaches themselves. “Did you notice any differences when answering the questions using the two different methods?” or “Which method did you use to better express your beliefs?” are examples of questions from the interview guide.

Steps two and three of the data collection process were videotaped so that the interviews could be transcribed and supplemented with notes on gestures (e.g., pointing to a card). To ensure pseudonymization of the data, personal codes were assigned to each participant to be able to relate questionnaire, Q sort, and interview later on.

### 3.3 Data evaluation

The theoretical two-dimensional structure of scales of beliefs on the teaching and learning of mathematics was already known from other studies such as TEDS-M or COACTIV (Fennema et al., 1990; Blömeke and Kaiser, 2010; Voss et al., 2013). Furthermore, the TEDS-M items in Table 1 have been used in other studies with pre-service teachers and the two-dimensional structure with the transmission-oriented scale on “learning mathematics through teacher direction” and the constructivist-influenced scale on “learning mathematics through active learning” (Laschke and Blömeke, 2013), has been confirmed on the basis of larger and comparable samples (Tatto et al., 2008; Buchholtz et al., 2013; Buchholtz and Kaiser, 2017; Yang et al., 2020). For this reason, it made sense to examine the factor structure in our study using a confirmatory factor analysis (CFA), which, however, did not yield results due to the small sample size and lack of prerequisites (Field et al., 2012). For this reason, only descriptive statistics were used to analyze the data set collected with the Likert-scale questionnaire, where mean scores of agreements with the items of the two scales proposed by the previous studies were calculated for each participant and correlated to obtain a measure of agreement with both scales.

The Q sorts of all participants were then statistically analyzed to detect different types of sorts. The evaluation of the Q sorts was done with the software KADE v1.2.1 (Banasick, 2019). In a first step, a Q correlation matrix was computed, representing the relationships between the Q sorts of participants. When applying Q methodology, researchers can use various factor analysis techniques, such as principal component analysis and varimax rotation, which aids in achieving more distinct and interpretable factors, making it easier to identify and describe the main viewpoints, or centroid factor analysis without rotation (which is less mathematically complex and aligns well with Stephenson’s original conception of Q methodology). The choice of method can, of course, influence the interpretation of factors. In this study, we used principal component analysis and varimax rotation in order to reduce the dimensionality of the data while retaining as much variability as possible and ensuring that each item loads strongly on one factor while remaining minimally associated with others. The selection of the number of factors was determined by applying the Kaiser criterion and looking at the Scree-test (Field et al., 2012; Moosbrugger and Kelava, 2012). The analysis yielded to three factors, as the eigenvalues showed a noticeable drop after the first and another smaller drop after the third factor. Finding three factors is common in Q methodology and reflects the method’s capacity to identify distinct yet interpretable viewpoints among participants (Lundberg et al., 2020). In the final steps of the analysis, the factor loadings were used to identify representative Q sortings of the participants for each factor (Banasick, 2019; Rahma et al., 2020). With the help of the software, the correlations of the factors were determined and the number of people with the respective prototypical sorting was estimated. In order to be able to make comparisons across factors, the weighted total score for each statement was standardized and converted into a *Z*-score (Watts and Stenner, 2012). The value indicates “how characterizing the statement is for the factor” (Sandhu and Hildebrand, 2022, p. 215). The *Z*-scores (in Q methodology also called factor scores) of the individual statements were used to create the prototypical sorting for each factor, which was then visualized by placing the items according to their *Z*-scores into the grid. Thus, each prototypical sorting shows a single Q sorting, which is also a very specific arrangement of statements that represents the perspective of the factor and is ideal-typical for it (Watts and Stenner, 2012). In this way, similarities and differences between the participants could be determined and individual types could be formed (Müller and Kals, 2004). On the basis of the calculated factor scores, statements were identified that were rated and placed very similarly (consensus statements) or very differently (distinguishing statements) by the participants (Sandhu and Hildebrand, 2022; Fisseler, 2023). Based on these statements, similarities and differences between the factors or types can be shown.

To enable the qualitative analysis, the 33 interviews were transcribed. The transcripts were analyzed using qualitative content analysis according to Mayring (2014, 2015), whose central element of analysis is a category system that abstracts the text structure on the basis of assigned codes. The software QCAmap (Fenzl and Mayring, 2017) was used to analyze and assign the codes. The categories were formed inductively from the material used but following the line of research questions RQ3 and RQ4. The following categories were used for the analysis

- Statements of reasoning on decisions to the positioning of statements in the Q sort (RQ3).
- General statements about the context of thought when deciding about where to put a statement in the Q sort (RQ3)
- Statements on the limitations or possibilities of the different approaches to capture or represent attitudes (RQ4)
- Statements about the preferences of either Q sort or Likert questionnaire (RQ4)

Instead of a quantitative count of the codes assigned, the analysis focused on a detailed consideration and interpretation of significant statements and patterns that emerged from the interviews. The “point of integration” in our study (Schoonenboom and Johnson, 2017) lies in the quantitatively driven qualitative evaluation of the interview data. Following our mixed methods approach, the qualitative data evaluation of the categories was guided by the explanation of the quantitative results of the Q sort analysis and was carried out descriptively on the basis of selected text passages that were representative of the previously identified distinguishing and most extreme statements of the prototypical Q sorts within the data material. Particular emphasis was placed on the depth of content and the specific perspectives of those participants of the sample that could be assigned the identified factors based on high factor loadings in order to ensure a nuanced understanding of the thematic diversity and complexity of the statements collected to each prototypical Q sort.

## 4 Results

In the following, we present the findings of our study along the lines of research questions RQ1 to RQ4.

### 4.1 RQ1: results from the Likert scale survey

The first research question (quan) focusses on the types of beliefs about the teaching and learning of mathematics that can be found among pre-service teachers using the Likert scales, and how they can be characterized. The results of the evaluation of the Likert questionnaire showed that our prospective teachers primarily have strong constructivist beliefs on average and the whole sample tended to disagree with the transmission-oriented items (see Table 2). However, possibly due to the small sample size, no significant manifest correlations were found between the two scales, so the results must be interpreted with caution. The results are in line with findings on comparable groups of students at other German universities (Buchholtz et al., 2013; Buchholtz and Kaiser, 2017).

**Table 2:** Sample means and range of participants’ agreement to Likert scale items.

Scale	Items	Mean	Min.	Max.	SD
Transmission-oriented beliefs	8	2.11	1.25	2.75	0.39
Constructivist-oriented beliefs	6	5.20	3.83	6.00	0.47

### 4.2 RQ2: results from the Q sort analysis

The second research question (QUAN) looks at the types of beliefs about the teaching and learning of mathematics that are found among pre-service teachers using the Q method, and how they can be characterized. The analysis of the Q sort revealed three different factors, i.e., typical sorts of a certain group of pre-service teachers. The factors explained 78% of variance in the sorts (*p* < 0.05). Of the 33 participants, 26 could be clearly assigned to one type. Nine people in the sample loaded on Factor 1, seven persons on Factor 2 and ten persons on factor 3. Seven people had strong secondary loadings and could therefore not be clearly identified. As Table 3 shows, the factors were strongly correlated, which indicates that the identified types differ only in nuances and the pre-service teacher present a rather homogeneous group concerning beliefs. Overall, there is a clear prioritization of constructivist statements and rejection of transmission-oriented statements in all factors.

**Table 3:** Correlation of the identified factors.

	No. of defining variables	Composite reliability	S.E. of factor *Z*-scores	Factor 1	Factor 2	Factor 3
Factor 1	9	0.973	0.164	1	0.7386	0.7497
Factor 2	7	0.966	0.184	0.7386	1	0.7242
Factor 3	10	0.976	0.155	0.7497	0.7242	1

**Figure 2:**
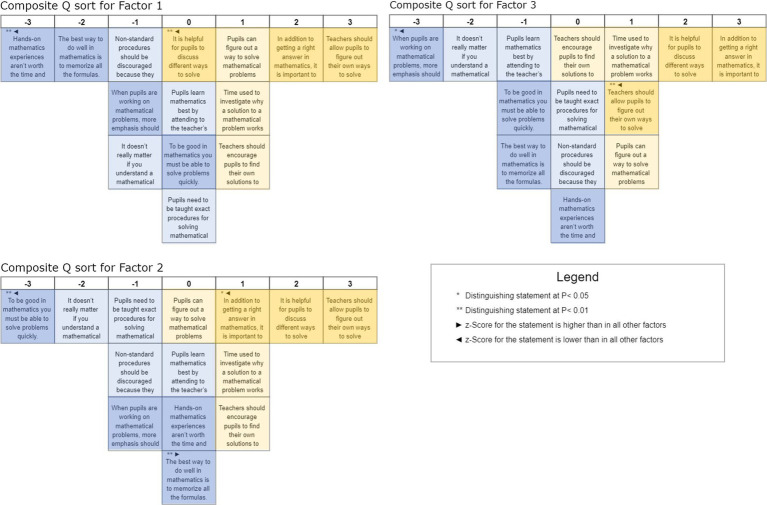
Composite Q sorts factor 1 to 3.

Figure 2 shows the prototypical Q sorts calculated in KADE. The statements are sorted in ascending order of their *Z*-Scores into the slots of an empty Q sort from −3 to +3. The statements that are significantly higher or lower in the individual factors than in the other factors, as well as the distinguishing statements that allow the interpretation of the respective factor, are marked in each case. In addition, we have colored the transmission-oriented (blue) and constructivist statements (yellow) for easier orientation; the consensus statements that did not allow any significant conclusions to be drawn about sorting patterns are colored lighter.

The following description of the three types is solely based on the prototypical Q sorts. The interviews were not taken into consideration at this point of time. Based on the sort-pattern, we were able to identify Type 1 *a posteriori* as an “understanding-oriented constructivist.” Having a more content-related view on the results we see in Figure 2 (top) that people of this type prioritize statement 8 “Teachers should allow pupils to figure out their own ways to solve mathematical problems” in the highest position followed by the statement 7 “In addition to getting a right answer in mathematics, it is important to understand why the answer is correct.” These statements are followed by various constructivist-influenced statements emphasizing the importance of giving students enough time in class to develop and explore their own solutions without a teacher’s help. However, this factor is distinguished by the neutral position of statement 14 “It is helpful for pupils to discuss different ways to solve particular problems.” A rejection of the avoidance of hands-on mathematics experiences due to time and expense (statement 10: “Hands-on mathematics experiences aren’t worth the time and expense”) is least important to these people. Statement 10 is the negation of a constructivist opinion. Thus, locating this statement in position −3 shows a strong denial of this negation and, thus, a strong acceptance of the underlying constructivist statement. This is followed by a strong disagreement on a one-sided focus on memorizing formulae.

We call Type 2 *a posteriori* “time-conscious co-constructivist” (see Figure 2, center). Like people in Factor 1, people of this type (tend to) agree with all constructivist-oriented statements, while they (tend to) disagree with all transmission-oriented statements. A more substantive examination of the results shows that people of this type also prefer statement 8 “Teachers should allow pupils to figure out their own ways to solve mathematical problems” like those of Type 1. People of Type 2, on the other hand, position statement 14 “It is helpful when students discuss different solutions to certain problems” in second place in terms of their agreement. They prioritize finding and discussing of own solutions even over getting the correct answer (statement 7). However, they emphasize nevertheless that it is important to understand why a mathematical statement is correct and that the time required for this is well invested. Statement 4 “To be good at math, you must to be able to solve problems quickly” and statement 3 “It does not really matter if you understand a mathematical problem if you get the right answer” receive the lowest level of agreement. This also shows a rejection of the focus on speed and correctness over understanding. Also noteworthy here is the neutral attitude towards memorizing formulas (statement 1), which are needed, for example, when finding your own solutions. This type emphasizes the awareness of the meaningful use of time in learning and teaching mathematics. People of this type believe that it is important to encourage students to develop their own solutions while discussing different approaches. At the same time, they emphasize that understanding different solutions and intensive engagement with mathematical tasks are more important than quick results.

We identified Type 3 *a posteriori* as “balanced process-product navigator” (see Figure 2, bottom). People of this type seem to value the exploration of different solutions and understand the importance of the process over simply arriving at the correct answer. Like Type 1, they show the (relatively) strongest agreement with statement 7 “In addition to getting a right answer in mathematics, it is important to understand why the answer is correct” but even more the (relatively) strongest disagreement with statement 6 “When pupils are working on mathematical problems, more emphasis should be put on getting the correct answer than on the process followed.” They also recognize the value in discussing various approaches (statement 14), however, they allow for personal discovery in problem-solving (statement 8) to a (relatively) lesser degree. Yet, they hold a neutral stance on the efficiency of solutions and the strict adherence to standard procedures (statements 2 and 9), and a skepticism about the usefulness and effectiveness of practical, hands-on experiences that deviate from conventional curricula (statement 10). This suggests a more balanced view that appreciates both the journey of learning and the flexibility in approaches, without a complete disregard for structure or efficiency. People of this type navigate between structured learning and the exploratory process, aiming to foster a deep understanding of mathematics through exploration and discussion of multiple problem-solving methods.

### 4.3 RQ3: results from the interviews about the Q sorts

The third research question (QUAN → qual) focusses on the insights on the types of beliefs from the Q sort that can be drawn from the different prioritization of items in the Q sorts with respect to the subjective evaluation of the items. In the following sections, results with respect to the three types are presented. The quotations used for illustration of the results were translated by the authors. Emphasis is marked in italics.

#### 4.3.1 Explaining type 1: “understanding-oriented constructivist”

With regard to the reasons for sorting statement 8 and 7, which were agreed with the most in relative terms, the subjective statements of participants assigned to Factor 1 show the high individual level of an orientation towards mathematical understanding and their belief of the importance of students finding their own solutions. The pre-service teachers of this type for example, judge this as very important for weaker students and in connection with the acquisition of problem-solving skills. With respect to their own teaching practice, this can generally also mean deviating from traditional approaches (16MAIG; 23SAEN).

“Yes, I think that [this] is simply a super important point in mathematics. Of course, that contradicts a bit of what I said earlier about [weaker] individual students who are better able to cope with being given a method to work with. But I think you should also give them the opportunity to find their own solutions first. And I simply believe that it’s really important for students to deal with problems in order to acquire these problem-solving skills, which they might be less likely to acquire if you simply provide them with solutions.” (16MAIG, factor loading 0.85).

“I then also thought a bit about my own learning groups and […] they were always happy to get their points for perhaps strange solutions that were nevertheless correct. But they were not necessarily happy to have to do it first because they were quite unsure about it. So perhaps this would have deserved a higher priority again so that the learners could become more confident. But in any case, I thought it was more important if they went their own way, not to sanction them in any way, but to say that if the way they went was right and not the one I had given them, then that was fine and I would accept it.” (23SAEN, factor loading 0.67).

At the same time, the individual justifications for the strongly rejected statements 10 and 1 reflect a collectively held constructivist belief of this type that successful mathematics learning goes far beyond the mere memorization of formulas. Again, the participants emphasize the great importance that understanding mathematical concepts and applications has for them. It is clear that a deep understanding of the subject matter and the practical application of mathematics are seen as essential for learning success (20SRRA; 23SAEN). In addition, the subjective value of practical experiences is emphasized (16MAIG; 20SRRA), as they help to better anchor what has been learned in the memory and deepen the understanding of mathematical concepts.

“If you memorize all the formulas, it does not mean you are good at math. It just means you are good at memorizing. I’m really against memorization.” (20SRRA, factor loading 0.69).

“And most of the time, these formulas give you prescribed ways to do it, the p-q formula or something. Sure, if you can memorize them, you can get through calculations quickly, but if you know what you are doing mathematically, then you can actually reach your goal quite well without this formula. And accordingly, in comparison with the other statements […], I found that it was no longer so important to simply be able to memorize the formulas, but to have understood the structure and to work with it.” (23SAEN, factor loading 0.67).

“‘Hands-on mathematics experiences aren’t worth the time and expense’. Yes, that’s another very extreme statement. That’s why it was easy for me to categorize it as very extreme in this case, because I think you can simply disagree with it. So especially in this area, I think time is never wasted […]. So, in this case I think it’s particularly important to gain these experiences, but basically experiences are always somehow worth the time and worth the effort.” (16MAIG, factor loading 0.85).

“No, practical experience is always linked to this, it stays in the head for longer.” (20SRRA, factor loading 0.69).

#### 4.3.2 Explaining type 2: “time-conscious co-constructivist”

Individuals of this type place higher value on allowing students to explore and find their own solutions, seeing personal discovery and the application of mathematical concepts as crucial to learning (statements 8). They prioritize the student’s personal autonomy in learning, and the co-construction of knowledge by discussion, valuing the individual’s process of understanding and the development of personal methods as more important than the mere acquisition of the correct answer. Moreover, both pre-service teachers 08SUDE and 19SULZ think that the discussion of various solutions already implies understanding a solution.

“So first of all, I think I found statement 8 more important than statement 14, because here the students simply approach the tasks individually themselves and filter out what they can do. Then this card [points to statement 14] is important because it’s a different skill to talk about [solutions]. So, for me, statement 8 was more the basic idea and, for me it actually reinforces the fact that you know why the approach is right. You know that automatically when you have found your own way. But why? So, for me, that [points to statement 8] is a bit of an overarching point that implies the other. And that’s why I thought it made sense.” (08SUDE, factor loading 0.84).

“That you should find your own way and give the students this opportunity. Because otherwise far too much is taken away in terms of discoveries or your own thinking and structure. Even if as a teacher you do not always understand what students are calculating, but as long as it works for them and it’s a constant that they can manage, then that’s worth the most to me, so it has to be on the far right side [+3].” (19SULZ, factor loading 0.84).

Pre-service teachers of this type strongly reject the notion that speed and the immediate correctness of answers are the main objectives in mathematics education (statements 4 and 3). This type believes that understanding mathematical structures and the ability to apply them thoughtfully are far more critical than being able to solve problems quickly. They are even frustrated by the educational focus on speed and express that understanding and describing mathematical processes should not be rushed, which is why they act time-consciously in their own teaching (11HERG).

“At first, I think I actually laid out the card with the formulas [statement 1], but then I thought about it. Okay, maybe you should know this one formula for percentages, even if you can perhaps derive it yourself, whatever. I was a bit hesitant at first, but then I swapped it, but then I was like, speed, if I somehow stress students or somehow want them to come up with a solution quickly. So somehow, I do not see any added value in that. Maybe that’s just the reality, unfortunately, if you do not have the time, but from my point of view, I would not want it. Maybe it’s just because you only have five minutes for a task because otherwise the lesson is over. So maybe there’s the counter-example of someone sitting at it for far too long, but it depends on whether they are just looking at it all the time or whether they are really still in a thought process and are just approaching a task, i.e., the solution. But I did not find the speed factor so relevant.” (08SUDE, factor loading 0.84).

“Because that’s what annoys me most about teaching math! […] It annoys me to no end that you always have to be fast in math […]. I do not understand why. It annoys me so much! […] Because I think that understanding and applying the structures of math and learning heuristics and things like that are much more important for the applications you want to do with it. […] All of that, that’s important, that I understand what’s happening and that I describe very well, but not that I describe quickly. So why do I have to be able to do it quickly? But it’s always like that in mathematical education. And that’s why I set it to ‘do not agree at all’, because it upsets me so much.” (11HERG, factor loading 0.53).

Interestingly, the “time-conscious co-constructivist” is neutral about the transmission-oriented need for memorizing formulas (statement 1), recognizing that while formulas can be helpful tools, merely knowing them is not synonymous with being good at mathematics (23UREN). They emphasize the importance of understanding how to apply formulas rather than just memorizing them, indicating a balanced view that recognizes the practical benefits of memorization but does not see it as the end goal (01BALN). However, this attitude also stems in part from the compulsion to assign transmission-oriented statements in the Q sort.

“The fact that I chose statement 1 was again a bit of a box-ticking exercise and you have to know the formulas, but you also have to understand how to apply them and just because I know formulas does not automatically make me good at math. I can memorize all sorts of things, but that does not mean that I’m good at it or really well educated or anything like that.” (23UREN, factor loading 0.84).

“Here I had far too much ‘do not agree at all’ and then I had to think about which of the things I can still get on with and I thought to myself, well, mathematics, that you have to memorize formulas, so it helps a lot that if you are at school and have to solve or prove something, maybe you remember certain formulas, I do not know, for example, for circles and cylinders, whatever. To be able to solve something in geometry, for example, formulas are an advantage. But I think … ‘*best* way … to memorize all the formulas’, that’s a bit, so … huh, I do not really agree with that, but it has to go somewhere that’s why I moved it to neutral.” (01BALN, factor loading 0.64).

#### 4.3.3 Explaining type 3: “balanced process-product navigator”

Like Type 1, the balanced process-product navigators express a strong subjective agreement with the idea that understanding why a mathematical solution is correct is paramount (statement 7). Depending on the mathematical content they personally value various solution methods as foundational to comprehending the rationale behind answers, like stated in statement 14 (06STEN). This is reinforced by the emphasis on the significance of grasping mathematical concepts and the process, rather than merely calculating the correct result (16BIEK).

“Because it is important to understand why a solution works or why the solution is correct and to understand the background knowledge and the result is of secondary importance, which is not so tragic, as long as you have perhaps understood the mathematical concept. It’s not good, but it’s more important to understand the concept behind it and the content than simply writing down a correct result. And, above all, to be able to justify why this result is correct and no other.” (16BIEK, factor loading 0.80).

“I think I’ve actually just said that I think it’s super important that the students are not taught that there is always this one solution only. There is only one approach and anything else is all wrong. That’s why I think it’s very important that they find their own way and maybe even figure things out on their own. Okay, there’s not just one procedure, but different possible approaches and that they can go through their own process without being told what to do and how to do it. Of course, it works better for some topics and worse for others, but I think it’s a very important approach. Definitely in the classroom.” (06STEN, factor loading 0.79).

Correspondingly, this type firmly rejects the notion that getting the correct answer should be prioritized over understanding the problem-solving process (statement 6). They distinguish between mere test performance and genuine learning, advocating for a comprehension-based approach to education that transcends rote memorization and applies to real-life situations. They also refute statement 3 and the idea that a single method should be used mindlessly without grasping the underlying mathematical principles (03SARG; 16BIEK).

“The fact that you do not have to have understood something, you just have to have come up with the right solution, that may be true at the point where you have to pass an exam. But in my view of education, that does not fit in. I would differentiate between the educational process in general and an examination situation, in the way that how examination situations are designed at the moment. I would say that the educational process in general is about enabling people to understand and solve problems and thus arrive at a solution, because whether the solution is right or wrong may be clear in many places in mathematics, but it is also not clear in all places. The reason why I no longer have statement 3 under ‘strongly disagree’ but under ‘disagree’ is that I thought that the result of statement 6 is important in the exam situation, but even there, students get partial points for the solution.” (03SARG, factor loading 0.77).

“Because I think – that’s also the fact that you somehow only apply one procedure and do not actually know what you are doing. This is almost exactly the opposite of statement 7, where you simply state the result, not even perhaps a calculation, but do not even know why this is correct and perhaps cannot even apply it to more complex situations, but simply do something without understanding. Somehow that does not have much to do with math. “(16BIEK, factor loading 0.80).

However, interestingly, there is a neutral attitude of people of this type toward non-standard procedures (statement 9). These individuals subjectively recognize the potential of non-standard methods in learning but also see the value in learning correct, standardized procedures initially (28ILEN), which is a more transmission-oriented view. Similarly, while they see the application of mathematics beyond textbooks as valuable, they remain neutral on the time and effort required for practical, hands-on experiences (statement 10), suggesting they weigh the relevance and accessibility of mathematics in the real world against the practicalities of such educational approaches (16BIEK).

“And, for example, non-standardized procedures should be avoided because they can interfere with learning the correct procedure, […] I think, when it comes to learning mathematics, for example, when learning a correct procedure, then a standardized procedure is really important at first. Using a non-standardized procedure at the end is also good, but in the middle or at the beginning could be really detrimental. So, in my opinion, you should really use the standardized procedure at the beginning and then take a non-standardized approach afterwards. That’s why it depends on what this statement means. That’s why I set it to neutral, which I thought was fine.” (28ILEN, factor loading 0.60).

“‘Hands-on mathematics experiences aren’t worth the time and expense’. That’s always a bit of a question: What exactly does hands-on experience or gaining experience mean? That’s why I did not actually find it that difficult here. […] I do not think I had that much of a problem with the neutral here.” (16BIEK, factor loading 0.80).

### 4.4 RQ4: results from the interviews about the preference of the method of survey

Finally, research question 4 ((quan + QUAN) → qual) looks at the insights on the preferences of the methods to survey beliefs (Likert scales vs. Q method) that can be drawn from the interviews. Based on the statements about the preferences of either Q sort or Likert questionnaire, in most cases (28 of 33), the participants in the study could be clearly assigned a preference for one survey method. Eight pre-service teachers favored the Likert questionnaire and 20 pre-service teachers preferred the Q sort when it came to describing which of the two survey methods they considered more suitable for expressing their beliefs towards the teaching and learning of mathematics. Five pre-service teachers rated the perceived advantages and disadvantages of the survey methods roughly equally and did not express a preference for a particular method. However, there was no systematic correlation with the assigned types, as can be seen in Table 4. It is interesting to note, however, that the majority of participants who were assigned to time-conscious Type 2 expressed a preference for the Q sort, i.e., the preference for an intensive but time-consuming examination of the statements was also evident here.

**Table 4:** Preference for the survey method.

	Likert-questionnaire	Q-sort	Undecided
Type 1	3	6	2
Type 2	1	7	0
Type 3	3	6	1
Unassigned	1	1	2
Total	8	20	5

The participants gave different reasons for preferring one or the other survey method. Based on their statements on the limitations or possibilities of the different approaches to capture or represent their beliefs, different patterns of justification were identified. Above all, the majority of participants found that the Q sort depicted the personal thoughts on the statements better and in a more differentiated way (24EVCK), mostly because the individual statements had to be placed in relation to each other during sorting and were not considered in isolation from each other (28ILEN). However, the higher degree of reflection was largely perceived as more challenging (08SUDE).

“Because [with the Q sort] you are forced to think about what is more important to you. I can theoretically tick ‘I completely agree’ everywhere on the questionnaire. But that has relatively little significance for you and for me, too.” (24EVCK, Type 3).

“The difference between the methods is that here [within the Q method] I have to compare the statements and also weigh up which statement is more important to me, or not so important, where I tend to agree, where I agree less. With the other questionnaire method, I have the freedom to weight many more things equally. On the other hand, with the sorting method you are more concerned with the statements and you compare them much more, which you do not do with the questionnaire, but with the questionnaire you can look at each question separately.” (28ILEN, Type 3).

“I think this method allows you to deal with things in a completely different way. You just do it more consciously. Because then I also imagined that when you ask me, why is this [points to card 7] more important to you than this [points to card 14], that I then have to have a reason for it. Somehow, both were somehow equally important to me. […] You just have to decide. But I always think it’s good when you are encouraged to self-reflect. That’s why I always find it really good for me, because I realize that I think I’m just too intuitive on the questionnaire. And that somehow makes [the Q Sort] more fun. It’s all somehow, as I said, in relation to each other. I tick here [points to the questionnaire] and have already forgotten the other one. Here [points to the grid] I’m exchanging ideas the whole time, thinking ‘Oh, I had that now, why is that more important to me now?’” (08SUDE, Type 2).

However, many participants also commented that the intensive cognitive engagement was related to the forcing of the mandatory structure of the Q sort, which was perceived both as stimulating (03SARG) and as a burden (05ERLG). In particular, the presence of a neutral area in the middle of the Q-sort, in contrast to the questionnaire, was also perceived as a key difference between the survey methods (11HERG), which was also perceived as challenging (24BERG).

“The questionnaire is more time efficient, but the fact that it was mostly congruent in the middle range and that only the extreme positions differed, I would say that the Q method was more suitable or represents a more accurate picture, not more suitable. I would say that this is generally true, that you open up the hierarchy, that you get more differentiated images, because you have to prioritize what is on the very outside. In my sort, the things that are further out follow on from the things that are further in the middle. It’s exciting to make connections. That can be productive.” (03SARG, Type 3).

“In the questionnaire I can give as many ‘I fully agree’ crosses as I want. […] What I definitely find a disadvantage here [in the Q sort] is that you have cards on the right for which you have no fields and you think to yourself, they have to go here, otherwise I have no space. You do not have that at all with the questionnaire. That’s a disadvantage here.” (05ELRG, Type 1).

“Here [points to the questionnaire] I was basically missing this neutral thing, which is why I was sometimes a bit unsure about statements. And then [points to the grid] with this neutral part I could also think again quite well ‘Okay, if I had felt rather undecided about the things’, by filling this neutral thing with a few things where I was sure, I then had to decide on a few things where I was also unsure, but still had to decide what is now more ‘agree’, what is more ‘disagree’. That’s somehow one thing that struck me here.” (11HERG, Type 2).

“I just found it very difficult with this neutral part, because I do not think I would have ticked ‘neutral’ so often. That, […] yes, […] I think that was the difference, that it’s difficult for me to see a ‘neutral’ at all anyway. So, for me, neutral means […] that I do not have an opinion on it, perhaps. And I would not say that about any of the statements. I think I have more ‘disagree at all’ or ‘disagree’ and ‘agree’.” (24BERG, Type 1).

## 5 Discussion

The integration of data proved invaluable in our study, particularly in the selection of cases for qualitative analysis based on quantitative results. This approach ensured that the qualitative interviews were informed by the underlying quantitative patterns, facilitating a deeper exploration of why certain beliefs were held by pre-service teachers. For example, the fine-grained analysis of types identified in the quantitative Q sort analysis was enriched by qualitative insights, revealing the underlying reasons for pre-service teachers’ prioritization of certain beliefs over others. This integration also enriches our understanding of pre-service teachers’ beliefs and allows for a more dynamic formulation of belief patterns with regard to the teaching and learning of mathematics. Our study contributes to a deeper understanding by moving beyond the static dichotomy of transmission-orientation vs. constructivism to accommodate the complex, sometimes contradictory nature of personal beliefs. This provides a deeper understanding of the complexity of personal beliefs with all their shades and facets. From a theoretical perspective, this enables us to understand more precisely why pre-service teachers have certain beliefs and which underlying frames of reference have led them to certain (dis)agreement of the facets of constructivist or transmission-oriented beliefs. If we understand these frames of reference more deeply and take them seriously, we might–as a practical implication of our study–find a starting point to influence the development of beliefs, for example in the context of teacher study programmes. Studies are needed to investigate a possible connection between courses focusing on certain elements from the background frames of reference and the development of pre-service teachers’ beliefs. In addition, further studies are also needed to analyse the relation between the differentiated belief types, the underlying frames of references and the teachers’ specific teaching practices in the classroom. In particular, the connection between beliefs, teaching practices and student performance is a promising field of mathematics educational and psychological research.

One of the critical achievements of integrating quantitative and qualitative methodologies in the mixed methods research design was addressing the blind spots associated with each. The Likert scale surveys, for instance, provided a broad overview, indicating a general preference for constructivist beliefs among pre-service teachers, but lacked the depth to explore the contextual and nuanced understandings that pre-service teachers hold. The Q sort, on the other hand, brought these subjective nuances to the foreground, highlighting individual variations in belief structures. This dual approach of using mixed methods allowed for a more comprehensive understanding of the pre-service teachers’ beliefs, highlighting both commonalities and variations that might be obscured if only a single method were employed. Thus, our study showed the richness of a mixed methods approach, yet, bearing in mind the rising amount of resources needed for the time consuming process of data collection and evaluation.

The identification of three factors in our study is partially consistent with, but also goes beyond, the constructs found in previous research. Jaschke (2018) identified a distinct constructivist type and a mixed type of transmissive and constructivist orientation in his dataset. The constructivist type consists of *n* = 14 pre-service teachers who agree more strongly with all constructivist-influenced items than with transmissive-oriented ones. Thus, there is a clear distinction between the items in the prototypical Q sort with a split Q sort grid into a blue (transmission-oriented items) and a yellow (constructivist-influenced items) half. In addition to this constructivist type, Jaschke (2018) also reports a mixed type (*n* = 6) in which there is no strict separation between the transmission-oriented and constructivist-influenced belief items. The number of individuals loading on each factor shows that in his sample, as in ours, the constructivist-influenced beliefs about teaching and learning mathematics seem to be stronger. We could not find such a mixed type in our data set. Yet, we were able to further differentiate between “understanding-oriented” and “time-conscious” constructivism. These distinctions revealed underlying practical orientations that were significant to the pre-service teachers depending on different contextual teaching situations and own teaching experiences, showcasing Q methodology’s strength in revealing typologies that are usually concealed in Likert scale evaluations. Additionally, the process of engaging with the Q sort was found to be particularly motivating for the majority of participants, suggesting that this method can enhance engagement and yield rich, reflective data–of course, at the cost of a time-consuming survey method and the participants forcing of sorting statements in an order. The findings nevertheless underscore the potential of mixed methods research in educational and psychological settings, particularly in studying complex constructs like beliefs. Q methodology, combined with traditional Likert survey techniques, offers a robust framework for capturing the rich, complex system of beliefs that pre-service teachers hold. As a methodological implication for further research, it also suggests a pathway for refining Likert-scale instruments to include more situation-specific items, potentially increasing their validity in terms of their sensitivity to the varied contexts in which teaching and learning occur. For example, our results could be used to develop additional items that reflect teacher beliefs on the teaching and learning of mathematics even better than only a set of 14 items, which has been used in many studies for reasons of economy, but has also been criticized for its validity (Aeschbacher and Wagner, 2016). Furthermore, this study’s approach could serve as a model for future psychological research into other domains of teacher beliefs or even extend into different fields where understanding the depth and diversity of personal beliefs is crucial. In general, we think that future research should also consider Q studies or–even better–mixed methods involving Q methodology to investigate other affective constructs that are influential for teaching or learning mathematics. Thus, a more fine-grained investigation of notions like mathematical identity, motivation, resilience, or self-concept might offer valuable insights to better understand teaching and learning of mathematics. We see even more potential in intercultural studies that might be able to better explain differences found in the data.

Despite these strengths, the study faced several limitations. First, the Q methodology does not refute the critiques often directed at the items used in such studies; it does not directly address potential biases or limitations in item design. As our results show, the 14 items used in many studies on mathematics teachers’ beliefs are quite suitable for mapping the beliefs of mathematics pre-service teachers, but our results also show that, based on the types identified, additional items developed in this direction could provide further insights. Moreover, the Q-sort process was time-intensive, which may limit its applicability in large-scale studies or under time-constrained conditions in general. Another challenge arises with participants who find little distinction between statements, as the forced distribution required in Q sorts can lead to artificial polarizations, potentially skewing results. Furthermore, Q methodology allows only for relative statements (e.g., “Statement X is more prioritized than Statement Y.,” *cf.* Jaschke, 2018), which can restrict the depth of absolute belief analysis. In this study, we counteracted this possible polarizations and shortcomings by presenting both Likert and Q sort survey formats to the participants and allowing them to express their preferences for one format and also any perceived bias. However, our results could still be biased by the selection of the sample, as we had to rely on a random sample from only two universities. Against the background of the partially contradictory findings to Jaschke (2017, 2018), it cannot be ruled out that this contributed in particular to the strong constructivist orientation of the sample and that we were therefore unable to identify any mixed types. The small sample size also presented significant challenges for conducting a confirmatory factor analysis (CFA). While the use of a validated survey instrument has helped to mitigate some concerns about the reliability and validity of the Likert scale data, it cannot completely overcome the limitations imposed by sample size. Researchers interested in using a combination of Likert scales and Q sort within a mixed methods study should therefore either interpret the results with due caution, especially when newly developed items are used, or use the results of the Q sort analysis to validate the Likert items. The use of the items within both Likert and Q research approaches had the advantage in our study that both approaches could be assessed by the pre-service teachers in the same way, so that their judgement related exclusively to the methodological survey of teacher beliefs. However, this configuration also means that the more in-depth types of beliefs could only be identified by applying Q methodology within the known range of items.

Despite these challenges, the integration of Q methodology with traditional Likert scales significantly enriches the research landscape. It allows for a more detailed description of subjective perspectives and provides empirical evidence of belief types that typically remain hidden in Likert scale evaluations. The qualitative feedback from the interviews added substantial depth, revealing the contextual nuances and expertise-driven motivations behind participants’ responses, which are invaluable for comprehending the complex terrain of mathematics teachers’ beliefs.

There has been considerable debate about whether Q methodology qualifies as a mixed methods approach (Newman and Ramlo, 2010). Q methodology inherently blends qualitative and quantitative techniques, making it a hybrid method. Stephenson, the founder of Q methodology, emphasized that it was designed to study both subjective and objective behaviors, rejecting the separation of objectivity and subjectivity (Stephenson, 1953). This integration is seen as aligning well with the mixed methods philosophy, which acknowledges the coexistence of multiple kinds of knowledge and combines qualitative and quantitative approaches (Johnson and Gray, 2010). Newman and Ramlo (2010) and Stenner and Stainton-Rogers (2004) have argued that Q methodology fits well within the mixed-methods continuum because it utilizes quantitative techniques like factor analysis to interpret qualitative data from Q sorts. This blend allows researchers to derive statistically robust patterns of subjectivity and subsequently interpret these patterns qualitatively, providing a comprehensive understanding of the research topic. Q methodology’s integration within the mixed methods framework is supported by its dual focus on qualitative insights and quantitative rigor. The methodology’s ability to provide context-rich, detailed descriptions while also allowing for statistical generalization makes it a powerful tool for mixed methods research.

## 6 Conclusion

This study utilized a mixed methods approach, combining quantitative Likert-scale surveys and Q methodology in a quantitatively driven concurrent-sequential (quan + QUAN) → qual research design (Morse, 1991; Schoonenboom and Johnson, 2017) to capture a comprehensive view of 33 pre-service mathematics teachers’ beliefs on the teaching and learning of mathematics. With regard to the Likert scale surveys, the quantitative results indicated that pre-service teachers tend to hold constructivist-influenced beliefs. The quantitative analysis using Q methodology furthermore identified distinct constructivist belief systems within our sample, delineating them based on their prioritization of statements. This was further enriched by qualitative insights from post-sort interviews, which illuminated the reasons behind participants’ prioritization of certain statements and provided context to these choices. In conclusion, we emphasize that while Likert-scale surveys can deliver broad insights across extensive samples, the Q methodology provides deeper insight into individuals’ subjectivity. Our results not only highlight the complexity of pre-service teachers’ beliefs about the teaching and learning of mathematics but also demonstrate the efficacy of integrating multiple research methods to capture this complexity fully. Such approaches are crucial in advancing our understanding of psychological phenomena and in developing more effective, context-sensitive instruments for measuring psychological traits, suggesting a promising pathway for future research in mathematics teacher education and beyond.

## Data availability statement

The datasets presented in this article are not readily available because access to the data or parts of the data is strictly limited and only accessible to researchers who are specifically authorized and have undertaken to comply with data protection regulations. The data was collected solely for the purpose of the study and cannot be used for other purposes. This ensures that the data is used exclusively for defined scientific purposes and that the integrity of the data is maintained. Requests to access the datasets should be directed to Nils.Buchholtz@uni-hamburg.de.

## Ethics statement

Ethical review and approval was not required for the study on human participants in accordance with the local legislation and institutional requirements. All data used in this study was completely pseudonymized. At no time was the data processed or stored in such a way that it was possible to identify the individuals involved. There was no risk to the privacy or rights of the individuals involved in the study at any time. The study was conducted in accordance with internationally recognized scientific and ethical standards, without using personal data in a way that would have required approval by an ethics committee. The studies were conducted in accordance with the local legislation and institutional requirements. The participants provided their written informed consent to participate in this study.

## Author contributions

NB: Writing – review & editing, Writing – original draft, Visualization, Validation, Supervision, Software, Resources, Project administration, Methodology, Investigation, Formal analysis, Data curation, Conceptualization. MV: Writing – review & editing, Writing – original draft, Visualization, Validation, Supervision, Software, Resources, Project administration, Methodology, Investigation, Formal analysis, Data curation, Conceptualization.
